# Ankle Myopericytoma: A Rare Case Report and Cytogenetic Study

**DOI:** 10.7759/cureus.21307

**Published:** 2022-01-17

**Authors:** Aqilah T Alqassab, Fatimah Z Alsadah, Tarek Elsharkawy, Mohammad Alhamad, Hassan Alsayed

**Affiliations:** 1 Pathology, King Fahd University Hospital, Dammam, SAU; 2 Pathology, Imam Abdulrahman Bin Faisal University, Dammam, SAU; 3 Orthopedics, Imam Abdulrahman Bin Faisal University, Dammam, SAU

**Keywords:** pdgfrb, myofibroma, pathology, ganglion cyst, myopericytoma

## Abstract

Myopericytoma (MPC) is an uncommon benign neoplasm of the skin and soft tissues belonging to a spectrum of tumors that are histologically recognized by their distinctive perivascular myoid cell differentiation. These distinct tumors are more prevalent among middle-aged males, and they arise more frequently in the subcutaneous tissue of the four extremities. In this paper, myopericytoma is reported in a 59-year-old Saudi male, presented with a painless small cyst involving the left ankle suspected clinically to be a ganglion cyst. Following surgical excision of the cyst, the diagnosis of myopericytoma was made based on the histopathological pattern of the disease. This paper focuses on the clinical and histopathological findings of myopericytoma and emphasizes the importance of immunohistochemistry as well as molecular testing in reaching the final diagnosis.

## Introduction

Myopericytoma (MPC) is a rare soft tissue neoplasm that is newly described as a separate disease entity in 1998 by Granter and his colleagues [1]. They proposed the name myopericytoma to label a spectrum of tumors that exhibit differentiation of perivascular myoid cells [1]. MPC is most commonly seen in males, with an incidence that is approximately two times more than that in females, and the average age at the time of diagnosis peaks around the fifth decade [2]. This tumor commonly develops as a slowly growing, painless, solitary mass of the subcutaneous tissues of the extremities with only a few cases arising in other sites [3]. MPC is distinguished from other tumors by its histological pattern. It is characterized by the multilayered growth of spindle-shaped myoid cells around the thin wall of the blood vessels. This distinct vascular characteristic is nonspecific and is demonstrated in a spectrum of other neoplasms, including myofibroma, endometrial stromal sarcoma, infantile hemangiopericytoma, leiomyosarcoma, angioleiomyoma, and glomus tumor. Therefore, immunohistochemistry is essential to assist in classifying tumors that demonstrate this vascular pattern [4]. MPC is usually considered benign with an innocuous clinical course, and only a limited number of cases are reported as malignant in the literature [2]. Standard guidelines regarding the management are still not available, and complete surgical excision of the affected area is the preferred and potentially curative method [5]. This paper reports a case of myopericytoma found in the anterior aspect of the distal left ankle in a 59-year-old Saudi male.

## Case presentation

A 59-year-old male presented to King Fahd University Hospital in December 2019 with a chief complaint of a solitary, small, painless swelling in the dorsal aspect of his left ankle for six months duration. The patient denied other clinical symptoms or changes in the shape or size of the swelling. He further denied any change in the color of the overlying skin and bleeding or ulceration of the mass. No constitutional symptoms such as fever, night sweats, anorexia, or cachexia were reported. Additionally, the patient’s past medical history was insignificant, with no previous surgeries. His physical examination revealed about 3 × 2 cm mass, firm, non-compressible, flexible, and mobile, with no overlying skin changes, and nontender upon palpation. His clinical findings led to the suspicion of a ganglion cyst of the left ankle; thus, the cyst was removed surgically, and the specimen was subjected to histopathology and molecular testing.

**Figure 1:**
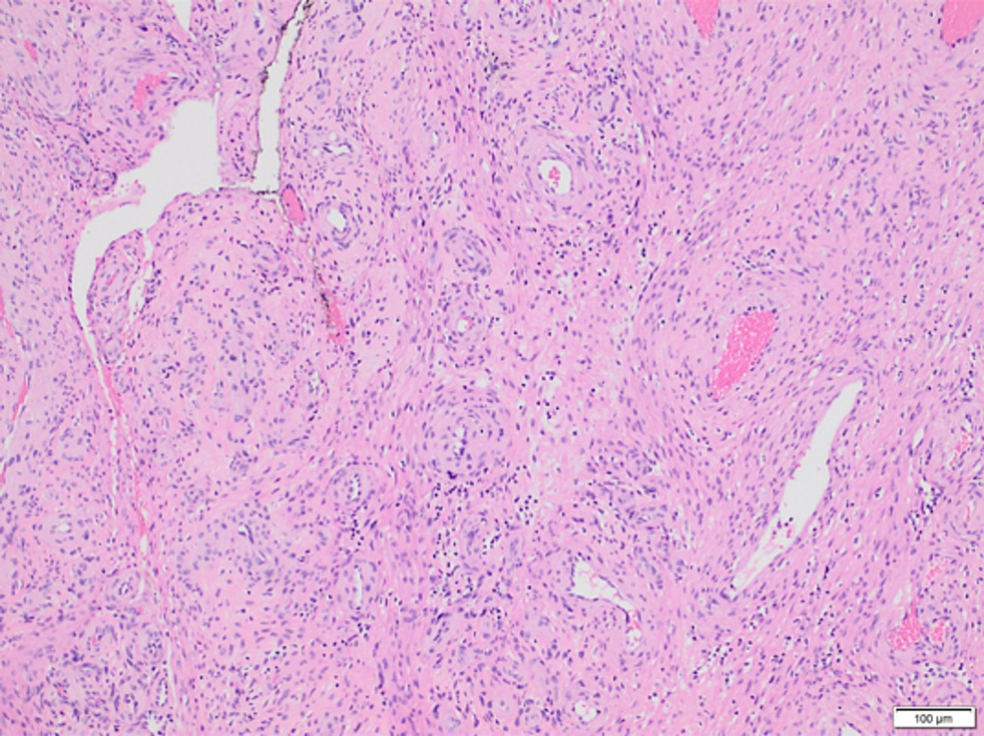
Low-power view showing nodular multilayered concentric arrangement of spindle cells around blood vessels (H&E, ×100).

**Figure 2:**
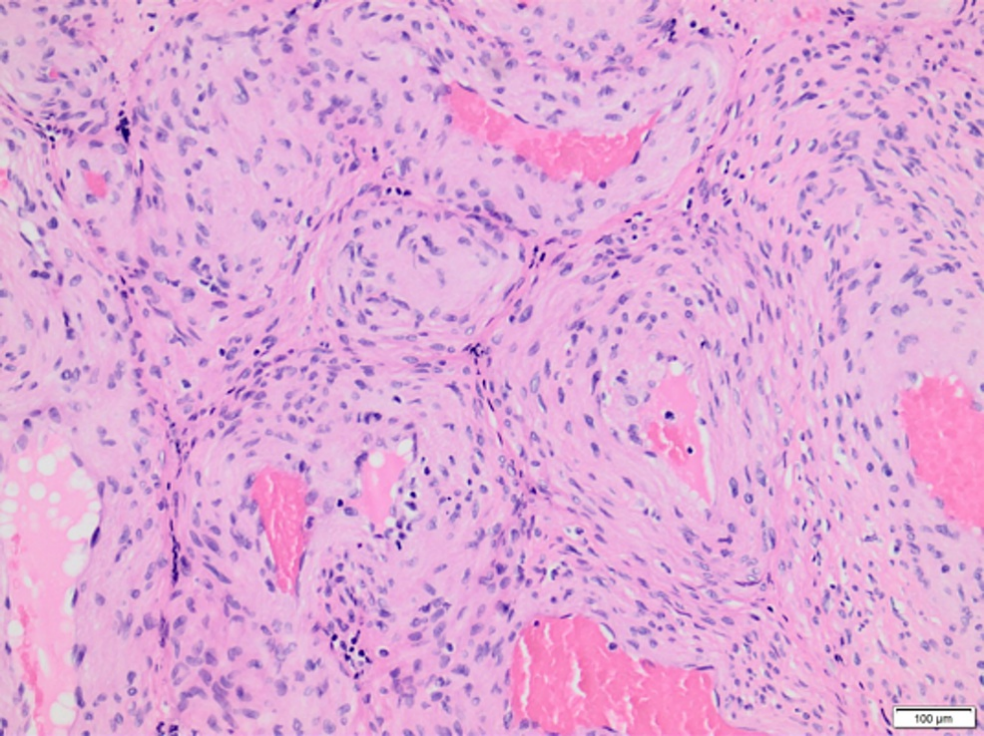
High-power view showing cellular and solid-appearing spindle-shaped cells with characteristic concentric arrangement around blood vessels (H&E, ×250).

**Figure 3:**
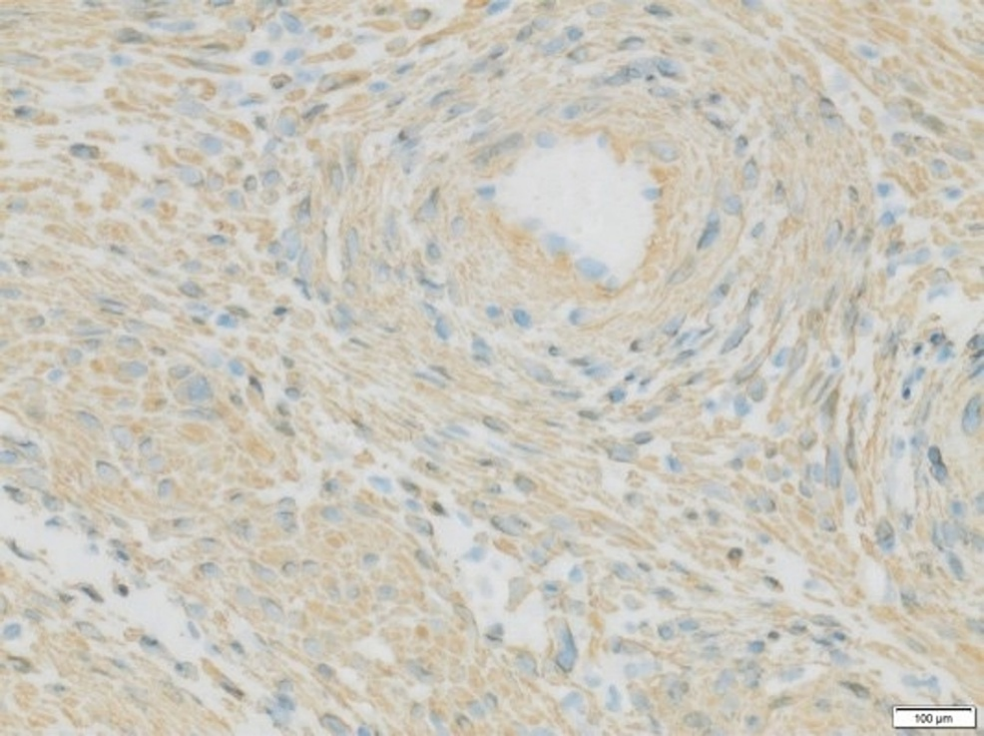
Positive immunostain with SMA in tumor cells (SMA stain, ×200).

The gross examination of the surgically resected specimen showed a single well-circumscribed cyst of white-tan, shiny, and soft tissue, measuring 1.5 × 1.3 × 1 cm. The cut section of the cyst revealed white-tan homogeneous parenchyma. Microscopically, the tumor was composed of spindle-shaped myoid cells with eosinophilic cytoplasm, showing concentric, multilayered growth around the blood vessels (Figure 1 and Figure 2). Immunohistochemical staining of the lesional cells showed appropriate reactivity for myoid origin: smooth muscle actin (SMA) was positive (Figure 3), whereas S100 protein and CD34 were positive only in dendritic and blood vessel cells, respectively. In addition, the tumor did not express human melanoma black-45 (HMB-45) nor desmin stains.

**Figure 4:**
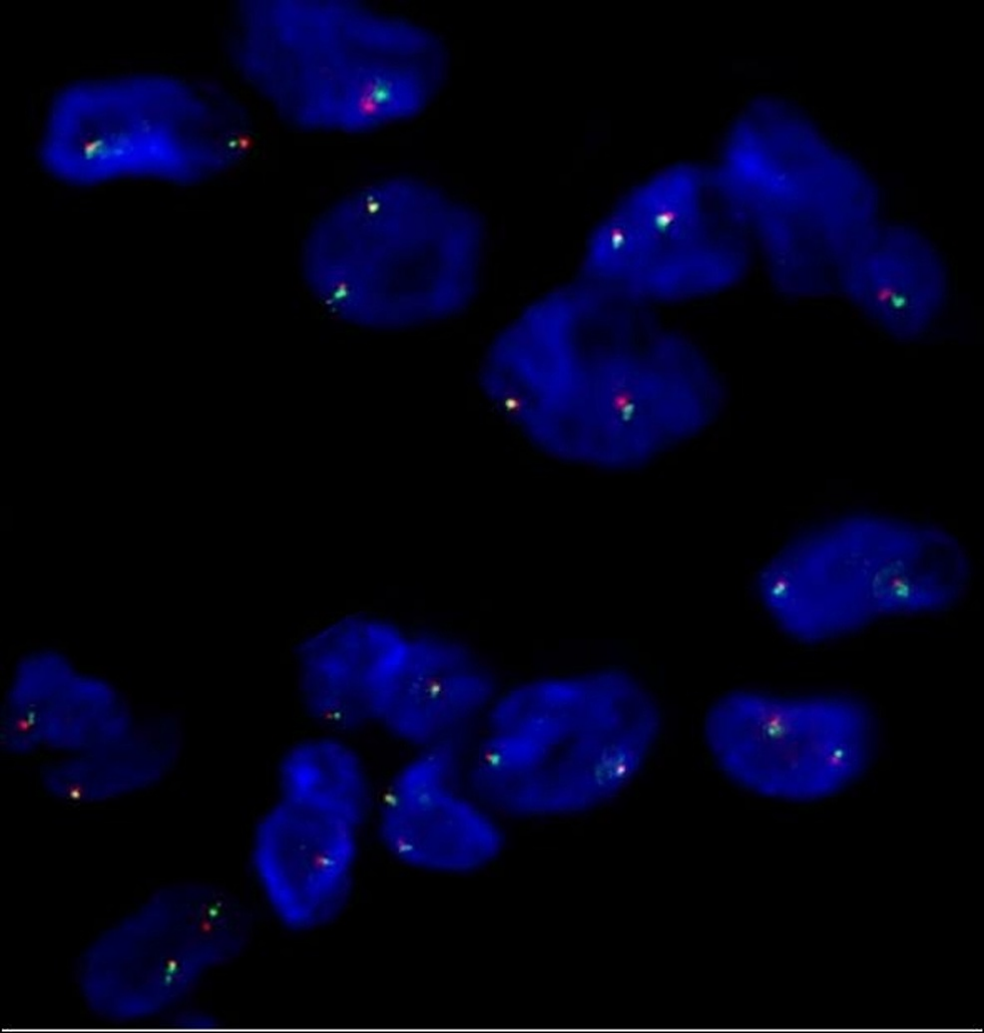
FISH of the dual-color PDGFRB break-apart probe. The tumor cells show normal two orange/green fusion signals.

As for molecular testing, fluorescent in situ hybridization (FISH) for platelet-derived growth factor receptor β (PDGFRB) break-apart probe was conducted on formalin-fixed paraffin-embedded (FFPE) tissue according to the manufacturer protocol (Abbott, Illinois, USA). The probes consisted of a dual-color, two probe mixtures of DNA sequences on specific regions of chromosomes 5q32-q33 gene regions. Signals were visualized under a Zeiss Axioscope microscope (Zeiss, Germany) using a fluorescein isothiocyanate (FITC)/rhodamine B (RhB) dual-band filter. The abnormal break-apart rearrangement probe involving the PDGFRB gene is one orange, one green, and one fusion signal. However, the expected pattern of the normal PDGFRB break-apart probe rearrangement is two orange/green fusion signals. The results of the FISH analysis of our specimen revealed normal expression (Figure 4).

The documented microscopic and immunohistochemical findings were highly suggestive of the rare variant myopericytoma. The patient was discharged from the hospital without complications on the same day of the surgery with a scheduled outpatient department (OPD) follow-up after two weeks.

## Discussion

Myopericytoma is a rare, slow-growing, benign tumor arising from the perivascular myoid cells and characterized by a hemangiopericytoma-like vascular growth pattern [6]. It was first proposed in 1992 to describe an unusual tumor in a young boy that displayed features between pericytes and vascular smooth muscle cells. Thereafter, in 1998, the term MPC was used formally by Granter and colleagues to classify a spectrum of tumors that demonstrate a striking concentric perivascular proliferation of spindle cells. In the 2013 World Health Organization (WHO) classification of soft tissue tumors, myopericytoma and other pericyte-derived tumors, including glomus tumors and variants, myofibroma, and angioleiomyoma, were listed under the pericytic neoplasm category [6-9]. Moreover, MPC can affect a broad range of ages, but it most commonly occurs in middle-age adulthood with known male predominance. The etiology of MPC is unknown; however, many factors were suggested in the literature that could have some association with the tumor, such as trauma on the affected site or viral infection, particularly Epstein-Barr virus (EBV) in patients with acquired immunodeficiency syndrome [10-12].

The majority of published cases reported tumors situated in the skin or soft tissue of distal extremities [2,6-8]. Less often, lesions arise at other sites, including the proximal extremities, head, and neck, and rarely visceral organs and intracranial sites [6]. A study that included 54 cases of MPC found that the tumor is most commonly seen in the four limbs, with the lower extremities affected more than the upper extremities, 26 and 16 cases, respectively [2]. This was followed by the head and neck region reported in only four cases, the truncal region in two cases, and exact location unknown in five cases [2]. The lesion usually manifests as a painless, slow-growing, well-demarcated, subcutaneous nodule. Although it often appears as a solitary lesion in adults, multiple lesions can be encountered, especially in pediatric patients [2,6,9]. The clinical presentation of the lesion appeared to be similar when it presents on different sites [2]. The patient in the presented case exhibited usual clinical findings as he is a male, and the lesion was located in a typical site, the lower extremity. In addition, the cyst was painless and fluctuating, with no signs of inflammation.

The diagnosis of MPC is usually established on the basis of histopathology and immunohistochemistry. In the case discussed in this paper, histopathologic features of the lesion were consistent with cutaneous MPC, and it was characterized by concentric perivascular proliferation of monomorphic, ovoid to spindle-shaped cells [6,7]. Furthermore, the average size of MPC in soft tissues is generally less than 2 cm, which is consistent with the diameter of the tumor in the presented case, which measured around 1.5 × 1.3 × 1 cm. Immunohistochemically, the tumor usually stains positive for SMA and h-caldesmon. On the other hand, it is nonreactive for muscle-specific markers such as desmin [9,13,14].

Differential diagnoses of this lesion are broad, and the diagnosis needs to be differentiated from myofibroma, hemangiopericytoma, glomus tumor, and perivascular epithelioid neoplasm (PEComa). With regard to myofibroma, it is controversial whether it is considered a subtype of MPC or a separate entity. Although it is composed of spindle-shaped cells with pale pink cytoplasm and arranged around the vessels, myofibroma shows a distinctive biphasic pattern [9,14]. Moreover, hemangiopericytoma rarely stains positive to SMA but shows consistent positivity for CD34 and CD99. On the contrary, MPC is almost always negative for these markers [9]. In the present case, CD34 was positive in blood vessels only. Glomus tumor can be distinguished by its distinct features under microscopy, including the presence of cells that are smaller in size with a rounded appearance, and the arrangement is in eccentric fashion around the blood vessels, with eosinophilic cytoplasm [7,9]. Finally, perivascular epithelioid neoplasm (PEComa), which is the least related tumor to MPC, can be differentiated by its morphological pattern in addition to its immunoreactivity to melanocytic and smooth muscle cell markers. Microscopically, it shows epithelioid cells with eosinophilic granular cytoplasm usually arranged in a nesting pattern. Immunohistochemically, HMB-45 is the most sensitive marker for PEComa, and it stains positive in approximately 100% of the cases. Other markers that stain positive include melan A, melanocyte-inducing transcription factor (MITF), desmin, and SMA [7,14,15].

Molecular testing has also contributed to the diagnosis of MPC. PDGFRB is a protein encoded by the PDGFRB gene that has been investigated in multiple studies to assess its implication in MPC. According to a study done in 2017, which concluded that among all genes tested in the study, namely, PDGFRB, BRAF, Notch homolog 1 (NOTCH), and glioma-associated oncogene homologue 1 (GLI1), PDGFRB alterations were identified in all cases of conventional MPC, while the rest came out negative [16]. In contrast, a study that analyzed PDGFRB mutations in infantile and solitary adult myofibromas, angioleiomyomas, and MPC showed dissimilar findings than those reported in the previous study. The mutations were detected in sporadic infantile myofibromas and adult myofibromas, in 75% and 69% of the cases, respectively. However, they were not detected in any of the cases of angioleiomyomas and MPC [17].

Surgical resection of the lesion is the procedure of choice for the diagnosis and treatment of MPC. Most cases behave in a benign fashion and have an excellent prognosis after complete resection. However, occasional local recurrences with incomplete excision and rarely metastases may occur in atypical and malignant neoplasms [6,14,16].

## Conclusions

Myopericytoma is an uncommon benign tissue tumor with a low rate of recurrence. Correct diagnosis and differentiation between the tumor and its mimics are essential to optimize patient outcomes. The combined use of immunohistochemistry and cytogenetics is useful to differentiate between similar lesions. Although molecular testing aids in reaching the diagnosis, the association between MPC and particular gene mutations is still inconclusive. Thus, the use of next-generation sequencing technology in studies with larger sample sizes is recommended in the future.
